# IoT System for Real-Time Posture Asymmetry Detection

**DOI:** 10.3390/s23104830

**Published:** 2023-05-17

**Authors:** Monica La Mura, Marco De Gregorio, Patrizia Lamberti, Vincenzo Tucci

**Affiliations:** Department of Information and Electrical Engineering and Applied Mathematics, University of Salerno, 84084 Fisciano, SA, Italy; m.degregorio19@studenti.unisa.it (M.D.G.); plamberti@unisa.it (P.L.); vtucci@unisa.it (V.T.)

**Keywords:** digital health, force sensing resistors (FSR), IoT, monitoring, posture, real-time

## Abstract

The rise of the Internet of Things (IoT) has enabled the development of measurement systems dedicated to preventing health issues and monitoring conditions in smart homes and workplaces. IoT systems can support monitoring people doing computer-based work and avoid the insurgence of common musculoskeletal disorders related to the persistence of incorrect sitting postures during work hours. This work proposes a low-cost IoT measurement system for monitoring the sitting posture symmetry and generating a visual alert to warn the worker when an asymmetric position is detected. The system employs four force sensing resistors (FSR) embedded in a cushion and a microcontroller-based read-out circuit for monitoring the pressure exerted on the chair seat. Java-based software performs the real-time monitoring of the sensors’ measurements and implements an uncertainty-driven asymmetry detection algorithm. The shifts from a symmetric to an asymmetric posture and vice versa generate and close a pop-up warning message, respectively. In this way, the user is promptly notified when an asymmetric posture is detected and invited to adjust the sitting position. Every position shift is recorded in a web database for further analysis of the sitting behavior.

## 1. Introduction

In recent years, outstanding growth of the Internet of Things (IoT) was enabled by the parallel progress of sensing technologies, miniaturization of electronic devices, cost reduction, widespread diffusion of consumer electronics, ubiquitous connectivity, and rise of cloud computing. Every technological advancement ideally has the ultimate goal of bringing benefit to humanity, hence the IoT paradigm unveiled the great potential of connected sensor systems for the improvement of the health conditions of all living being [1].

In this scenario of pervasive electronics, many IoT systems have been developed for monitoring vital signs and have later become commercially available. Examples include systems for the monitoring of the heartbeat [2], blood pressure [3], oxygen saturation [4], glucose level [5], breathing rate [6]. The monitoring of vital signs can be achieved by embedding sensors in wristbands, smartwatches, chest straps, or wearable apparel, and provides immediate feedback on the health condition of people that can be used either for anamnestic reasons or to trigger timely intervention in case of sudden events (e.g., heart attacks, strokes, suffocation) [7]. In the long term, health is strongly affected by environmental conditions, hence systems that monitor the air quality in terms of pollution and concentration of harmful particles also fall within the user-centered IoT measurement systems for human well-being [8]. In a broader context, considering the improvement of people’s general well-being, sensors, and IoT systems can be employed to monitor the perceived ambient conditions and eventually operate to set parameters, such as temperature and humidity, according to personal preferences [9,10,11,12]. Moreover, connected sensor systems also enable health preservation by doing environmental surveillance for safety reasons, for example, by checking constantly for the presence of smoke or toxic substances [13,14] or by performing structural health monitoring [15,16].

**Figure 1:**
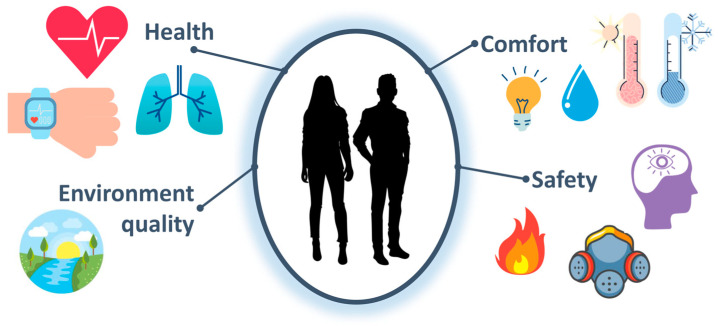
User-centered applications of IoT measurement systems for the improvement of human well-being based on the monitoring of vital signs and environmental conditions.

Figure 1 recaps the main examples of IoT measurement system applications designed to improve human well-being.

IoT measurement systems find application either in wearables or in smart homes and buildings, which include home and work environments. In wearable systems, sensors are embedded in clothes, fabrics, or accessories that the user carries on-body and are, therefore, suitable for monitoring vital signs and movement of all people. In home and work environments, sensors are hosted in the furniture or assembled in self-standing systems, therefore, they interact with the person either directly (e.g., sensors embedded in chairs) or indirectly (e.g., location systems detecting people’s presence in the room). Systems designed for home environment are generally aimed at the assisted living of elders. Systems designed for work environments target the preservation of the health and safety of workers. For example, in the context of smart homes and assisted living, Stern et al. [17] recently described a system for in-bed posture recognition by convolutional neural networks based on the pressure data acquired from a pressure mat positioned on the mattress aimed at investigating the relation between the sleeping positions and the insurgence of pressure sores in bedridden people. Leone et al. [18] developed a posture recognition machine learning algorithm for the classification of four postures: Standing, sitting, bending, and laying down. Concerning the monitoring of workforce health conditions, Lind et al. [19] overviewed the available systems based on motion capture instruments to collect kinematics data for the prevention of work-related musculoskeletal disorders.

Many jobs of the present time involve the use of personal computers for a long time. Typically, computer-based jobs require people to sit during work hours, enhancing the risks correlated to sedentary lifestyles. Among these, the problems related to a prolonged incorrect sitting posture are largely diffused and represent a health issue that many people around the world must deal with. The negative effects of prolonged bad sitting postures include musculoskeletal disorders involving the hip joints, lumbar area, upper back, shoulder blades, shoulders, and neck [20]. The condition of persistent loading on the intervertebral disks associated with poor postures is also responsible for the insurgence of low back pain [21], which affects people of all ages and is one of the main causes of absence from work [22]. Back pain is especially frequent in people with scoliosis, a pathological curvature of the spine that can cause chronic pain and prolonged asymmetric postures is, therefore, even more relevant for people who suffer from this condition. Although the incidence of scoliosis is not gender-related, it is found that female subjects are more likely to experience a progression of the spine curve [23]. The wide interest in posture-related health issues has originated several proposals for monitoring the workers’ sitting behavior.

In the past, different approaches to monitoring the sitting postures of long-time sitting workers were investigated in the context of the IoT applications for health risks prevention. Originally, several systems were proposed based on video analysis, though they never took place due to privacy issues. Then, textile sensors were largely considered for the development of smart fibers and wearables that could analyze the person’s posture by relying on dedicated posture classification algorithms. More recently, thanks to the widespread diffusion of low-cost and well-performing pressure sensors, the first solutions were replaced by sensor array-based systems.

In this paper, we describe a system for posture monitoring that falls within this category of sensor array-based systems. It is a portable system based on four pressure sensors that transforms a foam cushion into a ‘smart seat’ for the detection of the prolonged asymmetry of the worker’s sitting posture. The system is composed of the smart seat, a microcontroller-based acquisition system, Java-based software meant to run on the worker’s personal computer, and a password-protected web database for storing measurement data. The goal was to develop a system capable of detecting specifically the asymmetry of the sitting posture in real-time and provide timely feedback to invite the worker to adjust the sitting posture while also keeping track of the position shifts to inform following posture behavior analysis, all by using the lowest number of sensors and widely available electronic platforms to maintain low the overall cost.

The system in its preliminary form was presented in [24]. This paper extends the work in [24] by providing additional insight into the hardware and software of the posture monitoring system, providing more details on the asymmetry detection algorithm and code implementation, on the system capabilities, and on the opportunities for future developments. In this paper, we present a mature prototype of the posture monitoring system that addresses the challenges of performing an effective detection and notification of the sitting position asymmetry along the left-right direction while keeping a low cost and a low complexity from the point of view of technology and resource containment. The paper provides a detailed description of the system operation in terms of sensor data processing and management performed by the software, which implements a novel asymmetry detection algorithm.

In the following section, our work is framed within the state-of-the-art scenario of IoT posture monitoring systems by comparison with the most relevant works found in the literature on the same topic. In Section 3, the developed posture monitoring system is presented, discussing its main characteristics and capabilities. In Section 4, the hardware components (cushion, sensors, pressure adapters, read-out electronics) are described, and in Section 5, the software implementation and the asymmetry detection algorithm are explained in detail. In Section 6, an example of the software operation is provided. A discussion on the complexity of the system, the limitations of the system in its present form, and on the possible improvements coming from the addition of supplementary devices is reported in Section 7. Finally, the conclusions are summarized in Section 8.

## 2. Related Works

Before the explosive growth of embedded system technology and IoT, several implementations of sitting posture monitoring systems involved the acquisition of video recordings for an offline or real-time image analysis based on computer vision techniques [25,26,27,28,29]. These solutions were soon abandoned due to poor acceptability, as they required performing video recordings and for privacy issues. Thanks to the widespread diffusion and cost lowering of sensors, the easiest and more affordable way to assess the sitting posture is now based on pressure sensors.

Already in 2001, a ‘sensing chair’ for the classification of static postures was described [30], based on commercial pressure distribution sensors, allowing posture classification by exploiting pattern recognition algorithms already used in image processing techniques for computer vision.

In 2010, Meyer et al. [31] proposed a pressure sensing mat made of 240 sensing elements, capable of relieving the pressure applied over a surface by measuring the change of the capacitance between two electrodes made of conductive textiles and separated by a compressible dielectric spacer. The static pressure measurement was then used to recognize 16 sitting postures.

The eCushion presented in 2013 [32] is based on a textile pressure sensor made by placing on top and bottom of an eTextile (a composite yarn made of fibers coated with conductive polymer) fabrics coated by conductive buses. The posture monitoring system includes a cushion supplied with a 16 × 16 array of sensitive elements, an Arduino-based data aggregator, and a data analysis module. The seven-posture classification relies on a dynamic time warping-based algorithm. However, different weights, sizes, and sitting orientations impair the recognition algorithm performance.

Later on, in 2016, Zemp et al. [33] evaluated the accuracy of five different machine learning techniques for sitting posture classification based on a set of features extracted from 16 force and acceleration sensors applied to the seat pan, backrest, and armrests of an office chair. The goal was to analyze the sitting behavior, in terms of static position assumed during work, as well as of number and frequency of position shifts, in order to assess the level of comfort or discomfort perceived and eventually develop strategies for low back pain prevention.

The system proposed by Suzuki et al. [34] uses a commercial pressure mat made of a 16 × 16 pressure sensor array to generate a report about the sitting behavior, informing the user on the predominance of a particular sitting posture based on the distribution of the person’s weight on the chair seat. However, the report is generated after a sitting session, and therefore, does not provide real-time feedback on the assumed position.

In 2017, the smart cushion presented in [35] employed a 5 × 8 FSR sensor grid to recognize 15 sitting positions, although by working on the feature selection and the classification algorithm, Liang et al. managed to achieve good results by reducing the number of operated sensors down to 10.

More recent systems employ a reduced number of sensors and achieve a higher success rate in the identification of the sitting posture. For example, a system for the combined monitoring of the sitting posture and the electrocardiographic activity was proposed very recently by Pereira et al. [36], to recognize eight sitting positions by means of three load cells and monitor the heartbeat by means of conductive nappa leather dry electrodes, and the system proposed by Roh et al. in 2019 [37] identifies with excellent classification accuracy six different postures by using four low-cost load cells.

A system providing feedback on the assumed posture was proposed by Kim et al. [38] in 2018. The described real-time sitting posture correction system is based on textile pressure sensors, which return a different capacitance based on the applied pressure. The measurements acquired at 10 different points (six on the seat and four on the backrest) of the chair were used to identify seven different positions based on an ad hoc decision algorithm. The system is capable of providing real-time feedback on the sitting position by displaying posture changes on the PC monitor.

In 2021, Luna-Perejón et al. [39] proposed a six FSR-based system using a machine learning model based on artificial neural networks trained to identify seven sitting positions, i.e., the neutral correct posture and the six most frequent postures that can create issues in the locomotor system.

Anwary et al. [40] proposed an asymmetric posture detection system based on custom-made pressure sensors made from Velostat, a piezoelectric conductive material coupled to polyethylene foam and placed between two layers of conductive fabric. Four sensors embedded in the seat and two in the backrest provide the data to measure mild, moderate, and severe asymmetry of the sitting position based on a fuzzy logic algorithm. The system provides visual feedback of the sitting posture held during work hours by means of a mobile application, although it does not interrupt the user’s actions in case of prolonged severe asymmetry detection.

Nevertheless, despite all the previous works underlining the importance of keeping a correct posture while sitting for long hours, they focused on classifying the sitting posture and eventually providing feedback on the assumed sitting position without raising an alert to invite the user to correct their posture. In 2010, Hu et al. [41] proposed a wireless system for posture monitoring that provided a visual warning in case an inappropriate position was detected for a prolonged time. The system acquires pressure data from an accelerometer, two pressure sensors in the seat, and four pressure sensors in the backrest of an office chair (the number of sensors is limited by the microcontroller capabilities) and is managed by a mobile application that analyzes the sitting posture using a machine learning-based algorithm and sends the stored posture readings to a remote web server.

In the same year, Zheng et al. [42] proposed vibrotactile feedback for posture guidance. The developed system uses seven FSR sensors (five in the chair seat and two in the backrest) to recognize ten sitting postures and activates four vibration motors in case of bad posture, according to a pattern related to the incorrect posture assumed. In this way, not only is the user informed that the position needs to be adjusted but he or she is also guided through the position correction.

In order to understand which kind of warning is more effective and better tolerated, a study was proposed in 2011 [43], investigating different ways of notifying the sitting person about the harmful position held on the chair. The study involved the use of four force sensing resistor (FSR) sensors and had the only purpose of alerting the sitting person of the bad posture assumed by means of graphical, physical, or vibrotactile feedback. Among the considered modalities, the vibrotactile feedback proved to be more effective but also more intrusive, whereas the physical feedback was found sufficiently effective while being also less disruptive, and therefore, better tolerated.

Vibration feedback is included also in the work proposed by Ran et al. in 2021 [44]. In this case, like in [42], four vibration motors are activated according to a specific posture-related pattern to inform the user of the poor posture. In their work, the authors compare seven machine learning algorithms to classify seven different postures, using the data collected from a pressure sensing mat based on FSR sensors.

Table 1 collects the aforementioned works on sitting posture measurements found in the literature, highlighting the main features of each system proposed and exploring their capabilities and limitations, and compares this work to the others.

In this work, we adopt the same sensing technology (FSR) used in [33,35,39,41,42,43], which is cheaper than custom ad hoc-designed sensors as well as than other commercial solutions that provide better performance in exchange for much higher costs. In order to keep the cost low without impairing the efficiency, we employed the minimum number of sensors necessary to detect the posture asymmetry in an effective way, i.e., four sensors (as in [37,43]). The adoption of only three sensors (as in [36], where only one sensor is located in the front part of the seat) is, in our opinion, not sufficient for our goal of detecting the asymmetry in the left-right direction of both the front and the back part of the seat. Compared to other systems developed with the goal of recognizing a fixed number of standardized postures based on several different classification algorithms (e.g., pattern recognition and machine learning techniques), the system we have developed is aimed at the detection of the asymmetry of the sitting position along the left-right direction. In this aspect, the system we propose is designed with the same purpose as the systems described in [34,40,43]. Concerning the feedback about the incorrect sitting position, our warning is provided by an on-screen visual alert, as in [41,43]. However, with respect to the available studies that involve either similar technology, purpose, or alert system, our work differs in the approach to asymmetry detection. The novel algorithm we propose enables the reliable detection of the static asymmetry of the weight distribution in the left-right direction, while overlooking the acceptable transient asymmetric positions thanks to the moving-average filtering of the acquired measurements and allowing uneven pressure applied in the top-bottom direction to neglect the low-leg movements. Furthermore, our approach takes into account the uncertainty of the measurements and removes the issues caused by the unpredictable variability of the sensor response and provides real-time detection of the asymmetry and privacy-aware collection of the posture shift occurrences for further sitting behavior analysis.

## 3. The Posture Monitoring System

In order to keep the cost and complexity of the posture monitoring system constrained, we chose to employ four pressure sensors to recognize incorrect postures based on the comparison between the pressure applied to the left and right side of the seat. Aiming at developing a system that avoids the insurgence of back pain by inviting the worker to assume a correct sitting posture, we considered unnecessary the classification of a high number of different postures and focused on developing an algorithm for the assessment of the posture symmetry. In fact, in order to prevent back pain in the short term and other more severe issues in the long term, it is not necessary to recognize which posture the worker is currently holding. It is sufficient to verify that the assumed posture evenly distributes the weight force between the left and right side of the body. For this reason, the classification of the measured weight distribution in different postural figures is not relevant to determine the necessity to alert the sitting person.

**Figure 2:**
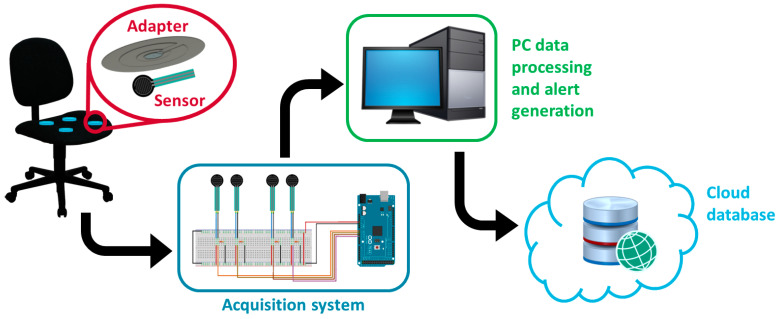
Schematic representation of the components and operation of the developed position monitoring system.

Figure 2 describes the components and operation of the developed posture monitoring system. The system includes four FSR sensors coupled to 3D printed PLA pressure scaling adapters and embedded in the seat cushion, positioned at the center of the four quadrants of the seat surface. The sensors are interfaced with a low-cost Arduino^®^ (Arduino, Turin, Italy) Mega microcontroller by means of a voltage divider, and the acquisition unit is connected by serial port to the personal computer, where a Java-based software processes the acquired data and implements the algorithm for the asymmetry detection and notification. The position shifts are recorded and stored in a password-protected database in the cloud, together with the user’s registration information.

**Figure 3:**
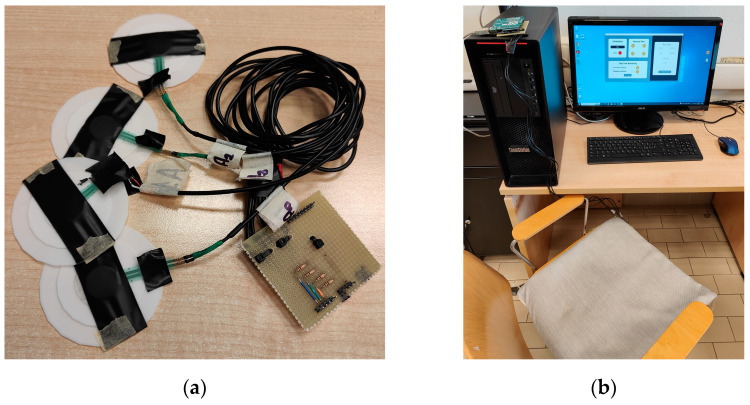
(**a**) Pressure sensors and acquisition system. (**b**) Mounted posture monitoring system in operation.

Figure 3a shows the sensors coupled to the 3D printed plastic adapters and the acquisition system, and Figure 3b shows the prototypal system in operation, where the sensors are placed in the cushion, and the acquisition board is connected to the workstation running the software program. During normal operating conditions, the acquisition system is housed inside a dedicated plastic box placed under the chair seat, and only the USB cable connects the chair to the PC.

The system performs an initialization of the pressure sensors to determine the reference measurement corresponding to the correct symmetric position, and monitors the posture symmetry in real-time by separately comparing the left and right sensor readings for the top and bottom sensor couple. When the posture shifts from a symmetric (correct) to an asymmetric (incorrect) position, the software prompts an alert that notifies the change and invites to switch back to the correct position on the chair.

**Figure 4:**
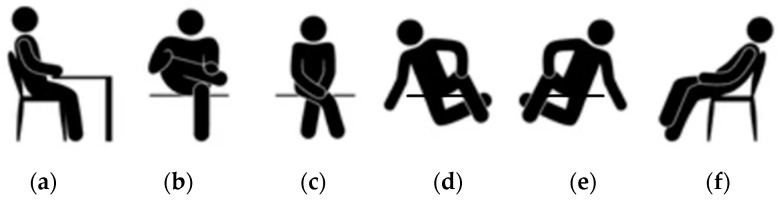
(**a**) Well-balanced symmetric sitting posture. (**b**–**f**) Asymmetric postures that trigger the system warning that invites the user to adjust their sitting position.

Figure 4 shows a collection of postures, one of which is well balanced (Figure 4a), and the others are detrimental to the spine health (Figure 4b–f) and trigger the asymmetric posture warning.

## 4. Hardware Components

### 4.1. Cushion

**Figure 5:**
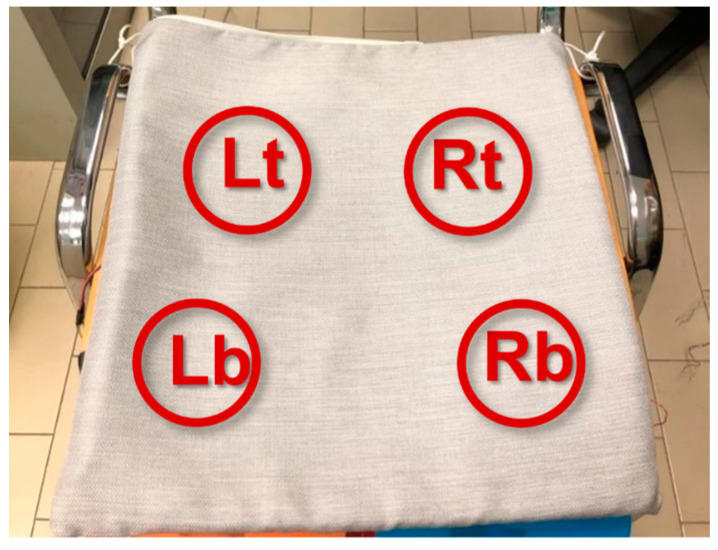
Pressure sensors’ positioning into the cushion. The four sensors are identified by Lt (**Left-top**), Rt (**Right-top**), Lb (**Left-bottom**), Rb (**Right-bottom**).

The sensors are placed under a 1 cm thick foam layer of square shape with a 40 cm side in top-left (Lt), bottom-left (Lb), top-right (Rt), and bottom-right (Rb) positions. The cushion protects the sensors and hides them from the user. The sensors’ positioning is sketched in Figure 5.

### 4.2. Force Sensing Resistors

**Figure 6:**
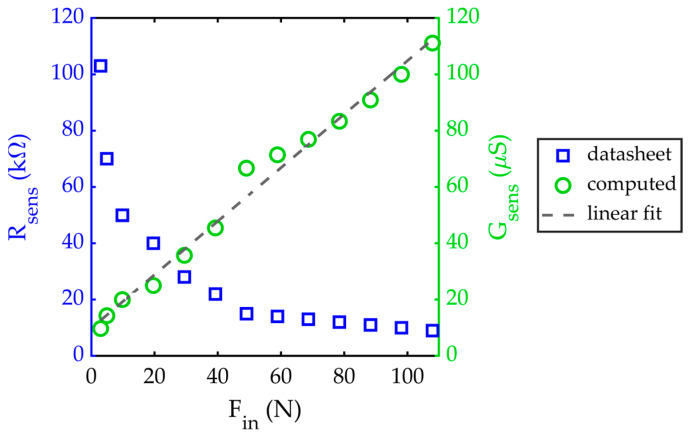
Calibration curve of the FSR pressure sensors used in the developed measurement system.

The sensors inside the cushion are four commercial force sensing resistors (FSR) DF9-40, cheap and well-performing. The sensors are made of a pressure-sensitive nanocomposite layer, which is a nanoparticle-filled polymer that, when pressed with an increasing applied force, increases the active conductive paths so that the overall resistance decreases. Thus, the sensor resistance R_sens_ inversely depends on the applied force F_in_, and the sensor conductance G_sens_ = 1/R_sens_ is approximately linear across the measurement range, as shown in Figure 6. The analytical relation between G_sens_ and F_in_ obtained by linear fitting of the calibration curve is:

G_sens_ = 0.950 F_in_ + 9.848 μS (1)

### 4.3. Pressure Scaling Adapters

**Figure 7:**
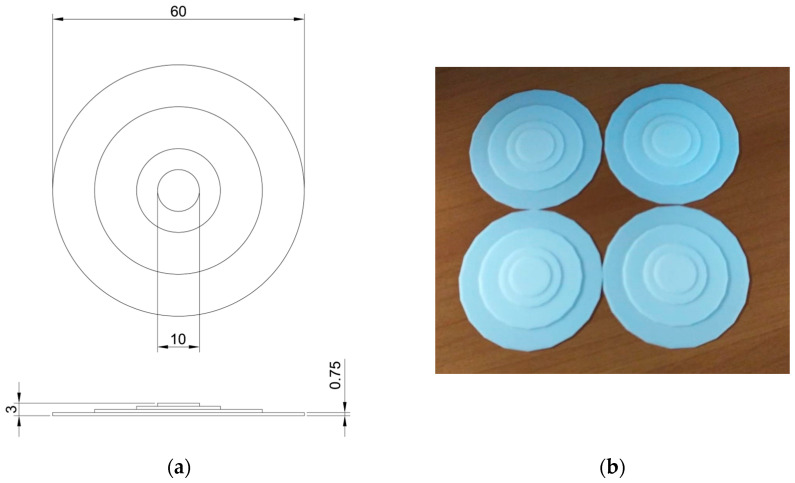
(**a**) PLA 3D printed pressure adapters and (**b**) their design.

Due to the very small sensitive area of the chosen sensors, we had to make some adapters to scale the force application surface down to the active area of the sensors. These adapters are made of poly-lactic acid (PLA) and realized by 3D printing superimposed circular layers with decreasing area. Figure 7a shows the design of the adapters and Figure 7b presents the fabricated disks. The PLA disks are then coupled to the four sensors and put on the lower side of the cushion, facing the chair seat.

### 4.4. Acquisition System

**Figure 8:**
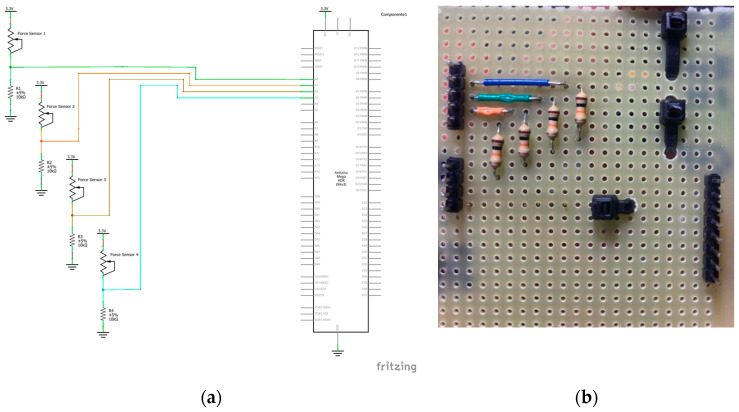
(**a**) Schematic and (**b**) picture of the acquisition system.

The read-out from the sensors is performed by acquiring the voltage on a known resistance in a voltage divider configuration with the sensor, digitalized by the 10-bit ADC of an Arduino^®^ (Arduino, Turin, Italy) Mega microcontroller board, and transformed into a force value according to Equation (1). Figure 8a reports the acquisition system schematic, and Figure 8b shows a picture of the acquisition circuit board.

## 5. Software Description

**Figure 9:**
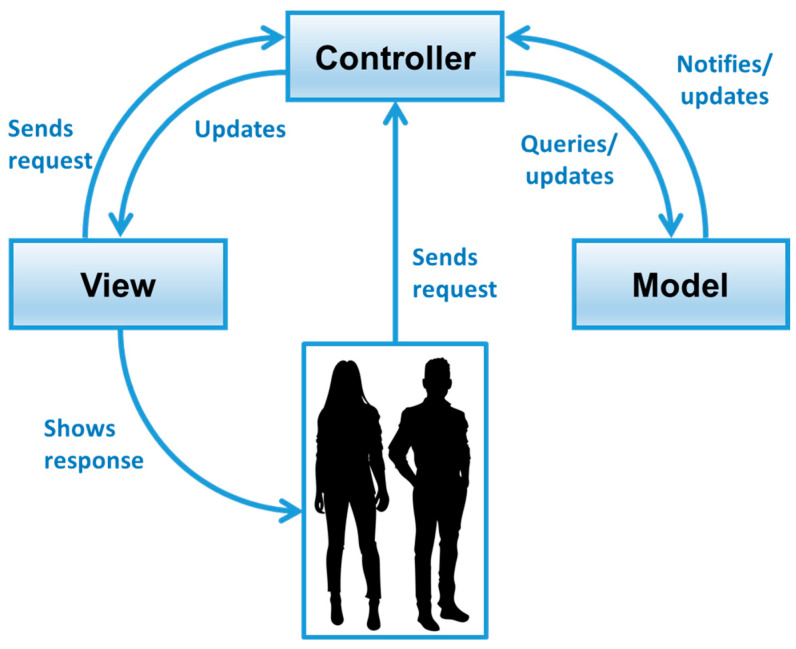
Interactions in the model-view-controller architectural pattern of the asymmetry detection and notification software.

The asymmetry detection and notification software is a Java-based multiplatform program designed according to a model-view-controller (MVC) architectural pattern. According to this pattern, the user interacts with the controller (i.e., the code managing the data flow) by providing the input force applied on the sensors and by operating the graphical user interface (GUI). The controller queries the model (i.e., the acquired data processing code) to retrieve the output of the measurements and the result of the detection algorithm. The model updates the controller by sending the elaborated data, hence providing the current status of the sensors and the symmetry/asymmetry of the posture. The controller also provides the data needed to inform the user about their current weight distribution on the smart seat to the view (i.e., the visualization of the current sensor status on the GUI). Figure 9 summarizes the interactions between the three actors of the MVC architectural pattern.

**Figure 10:**
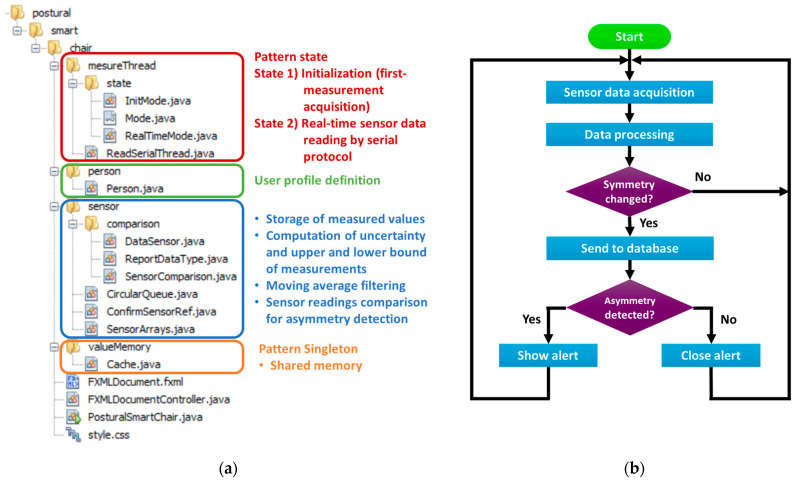
(**a**) Description of the software implementation: The code structure is reported, highlighting the function of each section of the project. (**b**) Workflow of the posture monitoring software.

In Figure 10, the structure of the software project is exploded, providing insight into the function of each section.

- The first section is the measure thread. The measure thread is written according to a ‘state’ pattern. The ‘state’ pattern allows changing the code’s behavior following the principle of the finite state machine. This means that, based on the current state, the program can make a transition to a restricted number of other states, and each state has different functionalities. In our case, the possible states are two: The initialization state and the real-time monitoring state.
  In the initialization state, the first acquisition from the sensors is performed. Due to the unavoidable differences between the response of the different sensors, this initial measurement works as a calibration by returning the center value of each of the four sensors associated with the correct position of the specific user.
  In the real-time monitoring state, the real-time sensor data acquisition is carried out with fixed timing.
- The second section includes the personal data collected for the user, defining the personal profile by listing the following tags: 1. name, 2. surname, 3. email, 4. height, 5. weight, 6. gender, 7. consent to data collection. The profile information is gathered for future statistical data processing, allowing disaggregated data manipulation, e.g., to perform gender-informed posture analyses. The user accepts the collection of the measurement data and of the personal information by ticking a checkbox. The consent can be revoked anytime by contacting the system administrator.
- The third section is dedicated to processing the data acquired from the sensors (filtering, uncertainty computation, upper and lower bound computation) and the implementation of the asymmetry detection algorithm (comparison between left and right measurement’s processed data).
- The last section defines the shared memory according to the ‘singleton’ pattern, which allows access to the instance from any part of the program without the risk of overwriting it.

The logic behind the software operation is described by the flowchart shown in Figure 10b. Upon launching the program, the software guides the user in assuming the correct position so that the reference pressure acquired from the sensors can be stored. Then, the software switches to the real-time monitoring state, running the acquisition and process of the measurements in continuous mode. The asymmetry detection algorithm outputs a ‘flag’ Boolean value indicating whether the sensors are measuring a symmetric or an asymmetric posture. As the flag shifts, i.e., a change has occurred from a symmetric to an asymmetric position or vice versa, the information is stored and registered in the database. In case the switch happens from a symmetric to an asymmetric position, a visual warning is prompted in the form of a pop-up alert to inform the user and invite them to switch back to the correct posture. If the switch happens from an asymmetric to a symmetric position, the pop-up alert closes autonomously. In this way, the database registers every position shift both towards an incorrect and a correct posture so that the time spent in a balanced and unbalanced posture can be calculated for each user, for example, in the view of generating a personalized report on the user’s sitting behavior.

Next, a detailed description is given of the sensor data processing and the asymmetry detection algorithm.

### 5.1. Sensor Data Processing

The read-out of the sensors is performed with a sampling time Δt = 500 ms. This time is long enough to allow the measurement acquisition by the microcontroller, the data transmission through the serial port, and the information processing by the personal computer to check for asymmetry.

**Figure 11:**
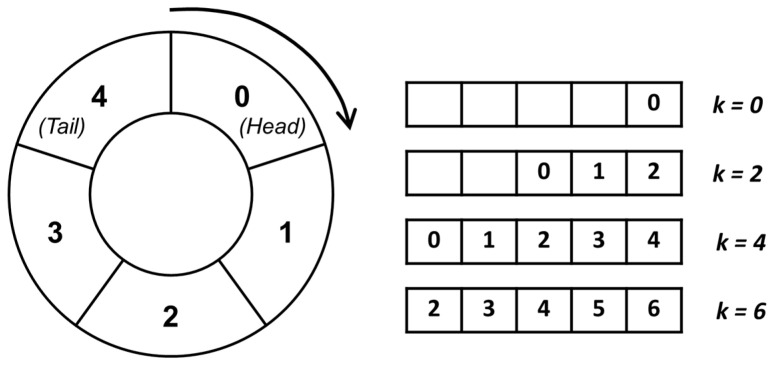
Representation of the 5-elements queue for the applied force value storage.

At the time instant t_k_ = k Δt, for each of the four sensors, the voltage V_m_ measured across the fixed resistor of known conductance G = 100 μS is transformed into the conductance value of the sensors according to the voltage divider equation:

G_sens_ = G·V_m_/(V_DC_ − V_m_) (2)

in which V_DC_ is the microcontroller DC bias voltage, V_DC_ = 3.3 V. G_sens_ is then converted into the applied force, F_in_, by means of Equation (1). The obtained input force value updates a 5-entry circular queue defined in the class ‘CircularQueue.java’. Every time a new measurement F_in,k_ is acquired, the oldest element queued F_in,k−5_ is cleared, and all the others shift one position to allow the latest measured value to enter the queue (see Figure 11).

The class ‘SensorArrays.java’ instances a 4-elements array, in which the four elements correspond to the 5-elements circular queue of the four sensors. Elements from 0 to 3 correspond to the top-left (Lt), bottom-left (Lb), top-right (Rt), bottom-right (Rb) sensor, respectively.

For all five values in each queue at the time t_k_, F_in,i_, with i = 0, …, 4, the type B measurement uncertainty is computed according to

(3) $$u_{k,i}=a_{i}/\sqrt{3}$$

in which a_i_ is the accuracy of the measured value as reported by the manufacturer on the datasheet, i.e., a_i_ = 2.5% F_in,i_.

The five values in the queue are then averaged, and the uncertainties propagated to compute the 5-point moving average of the applied force, obtaining the measured input force at time t_k_, F_in,k_^(m.a.)^ as

(4) $$F_{in,k}^{\left(m.a.\right)}=\frac{1}{5}\sum_{i=0}^{4}F_{in,k-i}\pm \sqrt{\sum_{i=0}^{4}u_{k-i}^{2}}=\langle F_{in,k}\rangle \pm u_{k}$$

The moving average filtering of the samples is necessary to perform low-pass filtering of the measured data, i.e., to neglect the effect of fast movements. In fact, the musculoskeletal disorders associated with sedentary work are related to the prolonged time of static loading on the intervertebral disks, whereas the dynamic movement of the spine and hips is not harmful to the sitting person’s health. For this reason, it is relevant to detect prolonged static asymmetric positions, neglecting short-term movements.

Starting from the moving average of the applied force, the upper and lower bound of the measured force can be computed:

(5a) $$F_{in,k}^{L}=\langle F_{in,k}\rangle -u_{k}$$

(5b) $$F_{in,k}^{U}=\langle F_{in,k}\rangle +u_{k}$$

Hence, for each sensor, the 5-point averaged input force and the uncertainty-related upper and lower bounds are known.

### 5.2. Asymmetry Detection Algorithm

The software intentionally compares the left and right measurements of the top and bottom couple of sensors separately. Hence, the user’s posture will be considered asymmetric both if the top couple or the bottom couple is unbalanced. Excluding the direct comparison between the top and bottom sensors allows accepting as correct postures all those positions in which the pressure applied on the top sensors is different from that applied on the bottom sensors, as long as the left-right balance is preserved. To further explain this aspect, it can be observed that the weight distribution between the top and the bottom area of the seat changes as the bending angle of the knee changes. This means that if the measurements of the top and bottom sensors were compared one with the other, the posture would be classified as asymmetric every time the person moves the legs closer or farther from the seat. Nevertheless, there is no evidence that uneven weight distribution along the top-bottom direction can generate a harmful loading on the spine, and it does not necessarily imply that the position is asymmetric also in the left-right direction. Hence, with the scope of revealing the asymmetry along the left-right direction only, the top and bottom couples of sensors are compared separately.

In order to ignore measurement discrepancies caused by the different response of the four sensors determined by the unavoidable variability of the sensors’ performance, the values of the input force insisting on each sensor are scaled by the first input force acquired during the initialization. In this way, every sensor’s reading is approximately equal to 1 when the user sits in the correct position. In the following, the 5-point average of the measured force of Lt, Lb, Rt, Rb, scaled by the 5-point average of the calibration force of the four sensors, is referred to as n-Lt, n-Lb, n-Rt, n-Rb.

**Figure 12:**
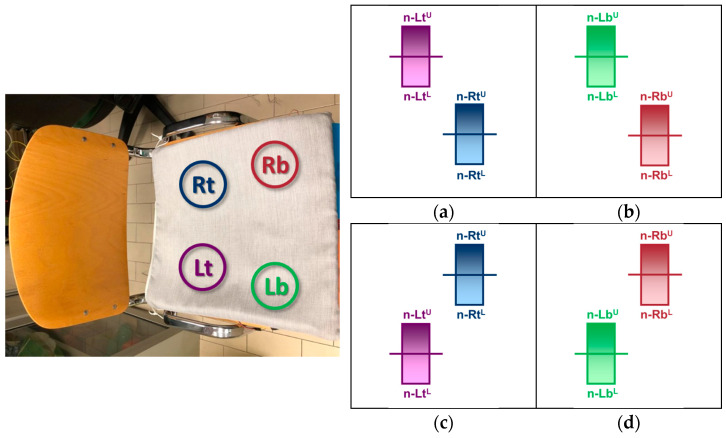
The four conditions that classify the sitting posture as asymmetric: (**a**) The lower bound of the top-left sensor reading is greater than the upper bound of the top-right sensor reading, (**b**) the lower bound of the bottom-left sensor reading is greater than the upper bound of the bottom-right sensor reading, (**c**) the upper bound of the top-left sensor reading is smaller than the lower bound of the top-right sensor reading, (**d**) the lower bound of the bottom-left sensor reading is smaller than the upper bound of the bottom-right sensor reading.

The applied force symmetry is assessed by comparing the upper and lower bounds of the left and right sensors, and the posture is considered symmetric if the uncertainty ranges of the left and right-side sensor measurements intersect. If the uncertainty range does not intersect, then the force applied on the left and right sides of the seat is uneven, and the posture is therefore considered asymmetric. Based on this consideration, there are four possible conditions that independently determine the asymmetry of the posture, visually described by the sketches in Figure 12:

1. The lower bound of the top-left sensor reading is greater than the upper bound of the top-right sensor reading (Figure 12a);
2. The lower bound of the bottom-left sensor reading is greater than the upper bound of the bottom-right sensor reading (Figure 12b);
3. The upper bound of the top-left sensor reading is smaller than the lower bound of the top-right sensor reading (Figure 12c);
4. The lower bound of the bottom-left sensor reading is smaller than the upper bound of the bottom-right sensor reading (Figure 12d).

The Boolean operation that returns a ‘high’ logic value when at least one of the different asymmetry conditions is true is the OR of the four comparisons:

(n-Rt^U^ < n-Lt^L^) OR (n-Rb^U^ < n-Lb^L^) OR (n-Lt^U^ < n-Rt^L^) OR (n-Lb^U^ < n-Rb^L^) (6)

The result of this comparison is stored in a variable that is updated at every time step. When the variable changes its value from 0 (‘low’) to 1 (‘high’), a shift from a symmetric posture to an asymmetric posture occurs. The information is recorded and sent to the web database, and a pop-up message warns the user that an asymmetry was detected. Conversely, when the variable changes from 1 (‘high’) to 0 (‘low’), a shift from a symmetric posture to an asymmetric posture has occurred. In this case, the position shift is also stored in the database. The alert window automatically closes.

The asymmetry detection algorithm was tested by two subjects intentionally assuming six incorrect positions and then restoring the correct posture. The six positions are: 1. leaning forward on the left, 2. leaning forward on the right, 3. leaning backward on the left, 4. leaning backward on the right, 5. crossing the left leg, 6. crossing the right leg. The same test procedure was repeated several times, and the algorithm always detected the position shifts correctly.

## 6. Software Operation

**Figure 13:**
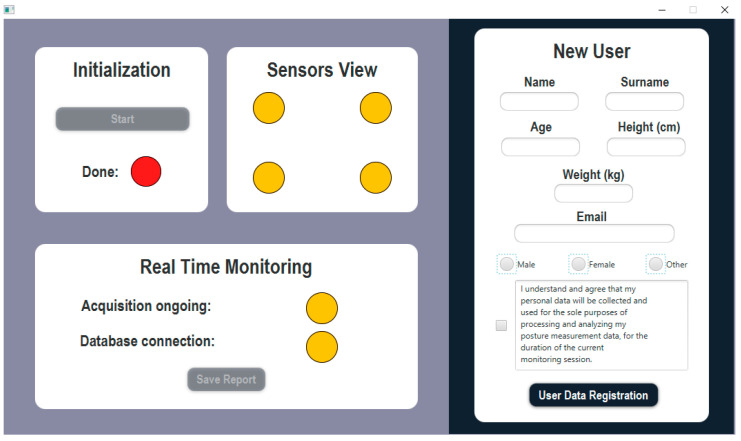
Posture monitoring software GUI.

Figure 13 shows the GUI of the software developed for the posture monitoring system. When the software is launched, the user fills the ‘New User’ tab on the right to register their personal information in the database. In accordance with the personal data management regulations, the user is also asked to check the box for consent to the collection and use of their personal data limited to processing and analyzing the posture measurement data for the duration of the starting monitoring session.

The first operation required is the software ‘Initialization’. During this phase, the sensors’ connection is tested, and the first measurement of the applied force is acquired. In this time, the sitting person is invited to assume the correct position on the smart seat. As soon as the five-elements queue is filled, the average force applied on each sensor is stored as a reference value. This operation serves as calibration because the following acquisitions from each sensor will be scaled by the reference value of the corresponding sensor. In this way, non-uniformities of the sensors’ response do not cause the erroneous detection of asymmetry. The initialization is performed every time the software is started, thus, every time a new monitoring session begins. In this way, the system is able to clear the impact of external factors on the sensors’ response, such as temperature and humidity or other environmental conditions variations happening in the time between separate sessions. The duration of each monitoring session can vary according to the worker’s national labor agreement, but it is generally advised not to maintain a sitting position for more than 2 h. Based on this consideration, we assume that the performance of the sensors remains unchanged within this time window. Once the initialization is correctly finished, the ‘Done’ indicator becomes green, and the ‘Sensors View’ tab becomes active.

The ‘Sensor View’ tab is purely informative of the correct operation of the four sensors and shows one indicator for each of the four pressure sensors embedded in the smart seat. The indicators are green when the read-out of the corresponding sensor is performed correctly, and red otherwise.

The ‘Real Time Monitoring’ tab is active immediately after the ‘Initialization’ is finished. The indicator of the ‘Acquisition ongoing’ is green when the real-time monitoring is running correctly, and red if there is some issue related to the connection with the microcontroller. The indicator for the ‘Database connection’ is green when the system is correctly capable of connecting to the web database to store the record of the position shifts, and red when the connection is broken (e.g., if internet connection is lost or the database server is not running properly).

**Figure 14:**
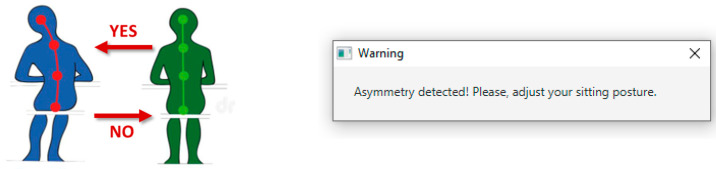
The pop-up warning generated on the PC screen when the shift from a symmetric to an asymmetric position, intentionally assumed during the algorithm testing, was detected.

When the shift from a symmetric position to an asymmetric position is detected, the system generates a pop-up message like the one shown in Figure 14. After the user adjusts the position improving the balance of the force distributed on the smart seat surface, and the shift from the incorrect position to the correct position is detected, the warning message closes autonomously. This alert generation and closing feature worked correctly during the asymmetry detection algorithm testing.

At the end of the monitoring session, the user terminates the software simply by closing the GUI window.

## 7. Discussion

### 7.1. Complexity of the System

The system we have presented is based on four FSRs. The number of sensors was chosen because it is the smallest number of sensors that allows detecting the asymmetry in the left-right direction both in correspondence of the hips section and of the upper legs. The benefits of using few sensors include an overall reduction of the complexity and cost of the acquisition system, as well as of the power consumption and computational effort required to run the system for several hours on a daily basis. In fact, increasing the number of sensors does not only raise the cost of the sensors. It also means using more powerful microcontroller units in place of the entry-level Arduino^®^ (Arduino, Turin, Italy) used in this system. Consequently, the power consumption would increase, and the software would become more articulated and computationally demanding. In its actual state, the system was designed to effectively detect the sitting posture asymmetry with the lowest complexity. Concerning the computational effort required to run the code, we assessed that the memory usage with the software running under Microsoft Windows^®^ 10Pro 21H2 (Microsoft, Redmond, WA, USA) on an Intel^®^ Xeon^®^ W-2245 CPU (Intel Corporation, Santa Clara, CA, USA) is comprised between ~150 and 160 MB of occupied RAM. In the next future, we plan to carry out a measurement campaign to have an estimate of the energy costs of the real-time monitoring per working day. This is aimed at upgrading the posture monitoring system by removing the cable connection in favor of a wireless transfer of the measurements, which also implies supplying the device with a rechargeable battery that can provide the energy required to cover an eight-hour workday. In this way, the smart seat will become stand-alone and fully portable.

### 7.2. Limitations and Accuracy Issues of the FSRs

If, on one side, the use of low-cost commercial FSRs allows achieving good accuracy while keeping the cost-constrained, on the other side, it comes with several drawbacks that cannot be neglected. First, commercial FSR sensors are accompanied by a datasheet with a calibration curve that may not be perfectly representative of the real response of each sensor due to the unpredictable variability of the fabrication process and the sensitive material performance. Anyway, performing an individual calibration of every sensor is not a feasible solution in the view of producing a commercial device on a large scale. Therefore, it is necessary to rely on the calibration declared by the manufacturer. To take into account the possible variability of the resistive response of the used sensors, we have decided to consider the measurement uncertainty during the comparison between the applied pressure measurements. Furthermore, the resistance value exhibited by the FSR sensors is easily affected by environmental conditions, such as temperature and humidity. Concerning the commercially available sensors, this often happens in an unknown way because the manufacturers do not always include such information in the datasheet of the product. In our case, we try to mitigate the impact of variations related to external factors by performing a new initialization of the sensors at the beginning of every monitoring session. In this way, the individual sensor’s resistance value that works as a reference for the correct posture reflects the sensor output in recent working conditions. This value is considered valid for the limited time of one monitoring session, which should last less than two hours (or however not more than the time indicated by each worker’s national labor agreement) and is reset periodically. Moreover, FSRs may undergo a progressive modification of their electrical response over time. To contain the possible effects of this response drift on the system performance, we have limited the validity of the reference resistance values to the duration of one monitoring session. For what concerns the long-time reliability of the sensors, the datasheet does not provide information about the mean life of the used devices. An assessment of the life duration of the developed system can be obtained only by performing future checks of its functionality. Finally, to investigate the reliability of FSRs for the proposed applications, further tests are planned to assess to what extent the positioning of sensors and the shape, ergonomics, and softness of chair on which the smart cushion is applied affect the functionality of the sensors and consequently the accuracy of the asymmetry detection.

### 7.3. Potential Improvements

Different options can be considered to improve the system.

An improvement in accuracy and reliability would follow the replacement of the low-cost FSR sensors with high-end force sensors. Sensitive elements with a wider active surface could increase the sensitivity, allow removing the pressure scalers and reducing the effect of the positioning of the sensors. Of course, this would determine a significant increase in the cost.

Although increasing the number of sensors on the seat would not affect the efficiency of the asymmetry detection, using additional sensors in other parts of the chair would extend the functionality of the posture monitoring system by enabling the detection of other relevant unhealthy postures. For example, prolonged asymmetric leg positions determine an uneven distribution of the static loading on the back and hip joints and could be detected by dedicated sensors. At the moment, we are embedding two sensors that measure the force exerted by the upper calf, related to the bending angle of the knee, with which we plan to monitor the symmetry of the leg position. Furthermore, we plan to add sensors in the backrest to monitor the symmetry of the trunk position. Since it is expected that the back does not stay in direct contact with the chair backrest for all the time spent at the workstation, a different sensing principle must be used to monitor the left and right side of the trunk distance from the backrest. One possible solution is the use of piezoelectric transducers to perform contactless distance sensing based on the time of flight measurement. The same type of sensors would also be employed to detect the distance of the head from the headrest. Combining the monitoring of the distance between the backrest and the headrest will allow the detection of leaning forward sitting positions, which are especially harmful for the neck and shoulders.

Finally, additional devices could be employed to make the asymmetry notification alert more effective or to let the user choose their preferred notification method. In particular, the system could be equipped with piezoelectric buzzers, like the ones already used in [45,46], to provide a sound alert, and with small motors, following the approach proposed by [42], to provide vibrational feedback.

## 8. Conclusions

IoT systems for monitoring workers’ conditions are starting to be greatly diffused due to the growing interest in the prevention of health risks in the workplace. Several musculoskeletal disorders are related to poor sitting posture held for long hours, which can happen to people involved in computer-based work, regardless of sex, age, and physical build. This work presented a system for real-time detection and notification of asymmetric sitting posture. Compared to other works found in the literature, the proposed system employs a small number of pressure sensors and microcontroller unit both low-cost and commercially available. The novelty introduced by the developed system stands in the asymmetry detection and notification algorithm implemented. The algorithm is uncertainty-aware and detects prolonged static asymmetry in the left-right direction for two observed areas of the chair seat (i.e., the top and bottom area) and is able to discard abrupt movements and fast position transitions. The system includes a multiplatform Java-based computer program that alerts the user with a visual warning message when an unbalanced position is detected. The visual feedback of incorrect position is embedded in the real-time monitoring software and is able to self-close when the correct position is restored. After acquiring the user’s consent to the management of personal data for posture monitoring purposes, the software records the worker’s position shifts in a password-protected web server so that further analysis of the person’s sitting behavior can be performed. Based on the personal information collected with the user’s consent, following gender-, age-, weight-, and height-informed analysis can be done to explore the sitting habits as related to specific characteristics. The workstations equipped with the presented system can support the improvement of workers’ health by preventing the low-back, shoulder, and hip joint pain related to prolonged poor sitting postures.

## Figures and Tables

**Table 1:** Overview of the posture monitoring systems based on pressure sensors found in the literature.

Reference	Year	N° of Sensors	Sensor Type	Posture Classification	Alert
[30]	2001	two 42 × 48	Commercial pressure sensing mat	yes	no
[31]	2010	240	Textile	yes (16)	no
[41]	2010	6	FSR	yes	yes (visual)
[42]	2010	7	FSR	yes (10)	yes (vibration)
[43]	2011	4	FSR	no	yes
[32]	2013	256	Textile	yes (7)	no
[33]	2016	16	MEMS, FSR	yes	no
[34]	2016	16 × 16	Commercial pressure sensing mat	no	no ^1^
[35]	2017	5 × 8 (10)	FSR	yes (15)	no
[37]	2018	4	Load cell	yes (6)	no
[38]	2018	10 (3)	Textile	yes (7)	no ^1^
[44]	2021	up to 44 × 52	Commercial pressure sensing mat	yes (7)	yes (vibration)
[39]	2021	6	FSR	yes (6)	no
[40]	2021	6	Velostat	no ^2^	no ^1^
[36]	2023	3	Load cell	yes (8)	no
This work	2023	4	FSR	no ^3^	yes (visual)

^1^ Gives feedback by visually displaying the acquired posture but gives no warning. ^2^ Recognizes correct posture and 3 levels of asymmetry. ^3^ Detects posture asymmetry.

## Data Availability

Data sharing is not applicable to this article.
